# Extracellular cold-inducible RNA-binding protein in CNS injury: molecular insights and therapeutic approaches

**DOI:** 10.1186/s12974-025-03340-7

**Published:** 2025-01-21

**Authors:** Dmitriy Lapin, Archna Sharma, Ping Wang

**Affiliations:** 1 Center for Immunology and Inflammation, The Feinstein Institutes for Medical Research, Manhasset, NY 11030 USA; 2 Zucker School of Medicine at Hofstra/Northwell, Manhasset, NY 11030 USA

**Keywords:** CNS injury, Traumatic brain injury, Ischemic stroke, Intracerebral hemorrhage, Neuronal death, DAMPs, eCIRP, Neuroinflammation, Efferocytosis, Therapeutics

## Abstract

Central nervous system (CNS) injuries, such as ischemic stroke (IS), intracerebral hemorrhage (ICH) and traumatic brain injury (TBI), are a significant global burden. The complex pathophysiology of CNS injury is comprised of primary and secondary injury. Inflammatory secondary injury is incited by damage-associated molecular patterns (DAMPs) which signal a variety of resident CNS cells and infiltrating immune cells. Extracellular cold-inducible RNA-binding protein (eCIRP) is a DAMP which acts through multiple immune and non-immune cells to promote inflammation. Despite the well-established role of eCIRP in systemic and sterile inflammation, its role in CNS injury is less elucidated. Recent literature suggests that eCIRP is a pleiotropic inflammatory mediator in CNS injury. eCIRP is also being evaluated as a clinical biomarker to indicate prognosis in CNS injuries. This review provides a broad overview of CNS injury, with a focus on immune-mediated secondary injury and neuroinflammation. We then review what is known about eCIRP in CNS injury, and its known mechanisms in both CNS and non-CNS cells, identifying opportunities for further study. We also explore eCIRP’s potential as a prognostic marker of CNS injury severity and outcome. Next, we provide an overview of eCIRP-targeting therapeutics and suggest strategies to develop these agents to ameliorate CNS injury. Finally, we emphasize exploring novel molecular mechanisms, aside from neuroinflammation, by which eCIRP acts as a critical mediator with significant potential as a therapeutic target and prognostic biomarker in CNS injury.

## Introduction

Brain injury harms the central nervous system (CNS) via both primary and secondary mechanisms [1, 2]. Primary injury mechanisms are diverse, from mechanical injury of the brain parenchyma in traumatic brain injury (TBI), to ischemia-reperfusion injury in ischemic stroke (IS) and intracerebral hemorrhage (ICH) [3–6]. The reported global incidence of TBI is 346 per 100,000 people, which may be an underestimate, making it one of the top causes of death and disability [7]. Similarly, the Nationwide Inpatient Sample (NIS) database reports the annual incidence of IS to be 236 per 100,000 hospital admissions in the USA, with a mortality rate of 3.1% [8]. For ICH, NIS data from 2004 to 2018 shows an average annual incidence of 23.15 per 100,000 admissions, with higher rates in recent years [9]. The estimated worldwide cost of TBI and stroke is massive, an estimated $861 billion and $400 billion, respectively [7, 10].

Secondary injury occurs through mechanisms such as ischemia, cerebral edema, elevated intracranial pressure, excitotoxic injury via glutamate release, breakdown of the blood-brain-barrier (BBB), and release of soluble inflammatory mediators including cytokines and damage-associated molecular patterns (DAMPs) [1, 3]. These inflammatory mediators hijack the innate immune system, both in the CNS and peripherally, to cause further tissue damage [1]. Moderate CNS inflammation in the acute phase is beneficial, while severe and dysregulated or chronic inflammation causes secondary injury [11, 12]. Importantly, even with focal injuries such as ischemic stroke resulting in the blockage of one blood vessel, secondary immune injury is global in nature. In murine models of stroke, sites that are distal to the infarct exhibit neuronal stress, apoptosis, and microglial activation [13]. Furthermore, imaging data from human patients with both IS and ICH has shown that microglia are activated in areas remote to the lesion and this activation persists for up to 6 months after the stroke [14].

We have discovered that extracellular cold-inducible RNA-binding protein (eCIRP) acts as a DAMP and is a novel mediator of neuroinflammation [15–17]. Indeed, eCIRP levels have been associated with increased severity and worse prognosis in patients with IS [18]. In this review, we will discuss the mechanisms proposed by which eCIRP worsens brain injury and explore eCIRP as therapeutic target in brain injury. First, we will provide an overview of neuroinflammation in brain injuries with a focus on eCIRP and its release in brain injury. Next, we will review the proposed mechanisms through which eCIRP affects variety of CNS cell types in brain injuries. We will then analyze the prospects of therapies that target eCIRP aimed at attenuating neuroinflammation. Lastly, we will evaluate eCIRP’s potential as a biomarker for brain injury.

### Neuroinflammation in CNS injury

Various types of cells can release soluble inflammatory mediators in CNS injury via different mechanisms. Microglia, the CNS resident immune cells, and infiltrating peripheral immune cells such as neutrophils, macrophages, and T cells have been shown to release pro-inflammatory cytokines [19]. Both in animal models of stroke and human IS patient CSF and blood samples, cytokines including IL-6, TNF-α and IL-1β have been shown to be elevated and released [19]. DAMPs, also known as alarmins, are signaling molecules released by stressed, and dead or dying cells that activate the innate immune system by binding pattern recognition receptors (PRRs) [20, 21]. In fact, multiple DAMPs have been well established as promoters of cytokine release through various mechanisms. ATP is known to bind to the P2X7 receptor resulting in pore formation and IL-1β release [22]. S100B, a DAMP mainly expressed by astrocytes in the CNS, can bind RAGE expressed on microglia and activate NF-κB to promote transcription of cytokines, thus upregulating expression, and release of IL-1β and TNF-α [23]. Similarly, HMGB1 induces phosphorylation of NF-κB and the release of pro-inflammatory cytokines IL-1, IL-6, and TNF-α via TLR4 signaling [24, 25]. Clearly, a variety of DAMPs promote cytokine production and release. DAMPs directly signal multiple cell types in CNS injury. RAGE activation by HMGB1 and S100B has been shown to cause BBB dysfunction in TBI via pericyte migration or loss, thus exacerbating hippocampal edema and behavioral deficit [26]. In a murine IS study, inhibition of HMGB1 with 18β-glycyrrhetinic acid nanoparticles promoted M2 microglia polarization and reduced M1 polarization along with infarct volume and behavioral deficit [27]. Similarly, in a middle cerebral artery occlusion (MCAO) IS model, S100B was found to promote microglial expression of M1 markers while suppressing M2 markers, and exogenous administration of recombinant S100B increase infarct volume in a dose-dependent manner [28]. It is important to note that these findings are not without limitations, as they utilize the canonical M1/M2 classification, which does not encompass the vast heterogeneity of microglia in various disease states [29]. In the present review, M1 and M2 phenotypes originally reported in the cited literature will be described as “pro-inflammatory” and “anti-inflammatory” microglia. P2X4R is upregulated in IS, and it is known that microglia express P2X4R and respond to ATP by becoming more amoeboid and producing pro-inflammatory cytokines [30]. Furthermore, pharmacologic inhibition of P2X4R in a model of ICH resulted in reduced neuron loss, BBB permeability, and neurological deficits [31]. Although it is highly important, the landscape of DAMPs and their potent effects in CNS injury is too broad to be reviewed herein. In summation, DAMPs released in brain injury are known to act on various cell populations, and mediate cytokine/chemokine signaling to aggravate CNS injury.

### eCIRP exacerbates CNS injury

CIRP is a constitutively expressed small RNA chaperone protein, composed of 172 amino acids, that under steady state conditions resides in the nucleus and functions to regulate mRNA translation [32, 33]. Hypothermia enhances CIRP expression, an important protective mechanism for cells to respond to such environmental changes [34]. Several studies examining intracellular CIRP (iCIRP) in the context of CNS injury have shown a neuroprotective effect. Mild hypothermia resulting in iCIRP induction inhibits apoptosis via ERK-1/2 activation to alleviate injury in TBI [35]. iCIRP overexpression in neurons downregulates hypoxia-inducible factor-1α (HIF-1α) expression and HIF-1α-mediated neuronal apoptosis in hypoxic-ischemic brain injury in neonatal rats [36]. Another recent publication reported that neuron-specific overexpression of iCIRP in the hippocampus attenuated excessive iron-induced ferroptosis and memory impairments in the offspring in a model of pregnant mice exposed to hypoxia [37]. Overexpression of iCIRP was also shown to improve cell viability and upregulate EGF expression in glial cells, implying glial EGF-activation mediated neuroprotection [38]. In contrast, various types of cellular stress can cause the translocation of CIRP from the nucleus to the cytoplasm and ultimately to the extracellular compartment, resulting in extracellular CIRP (eCIRP), which we have shown to be an inflammatory mediator, unlike iCIRP [15, 39].

eCIRP has also been shown to move into stress granules, or accumulating translation initiation complexes of protein and RNA, that can either disassemble if the stressful stimulus abates or be released extracellularly via exocytosis if the stimulus persists or worsens [40–43]. We recently discovered another mechanism for active release of eCIRP, via formation of Gasdermin D membrane pores in septic macrophages, which occurs in a pyroptosis-independent manner [44]. Necroptosis, an orchestrated form of cellular necrosis, is a recently discovered mechanism of eCIRP release, specifically via macrophage necrosome activation and subsequent phosphorylation of mixed lineage kinase domain like pseudokinase resulting in pore formation on the cell membrane though which eCIRP can escape [45]. Multiple active mechanisms for eCIRP release, recently reviewed in detail, have been discovered and may potentially act to increase eCIRP in CNS injury [46]. However, the role of necrosis-mediated passive release of eCIRP cannot be discounted due to the widespread necrosis known to occur in CNS injury [47–49]. We have shown that BV-2 murine microglial cells upregulate and release eCIRP under hypoxic conditions, strikingly upregulating eCIRP mRNA up to 10-fold and protein levels > 20-fold in culture medium compared to normoxic controls [50]. Furthermore, alcohol and amyloid upregulate release of eCIRP into cell culture medium of BV-2 cells [16, 51]. Interestingly, neuron-derived eCIRP also plays a role in CNS injury; with a recent study showing primary neurons subjected to oxygen-glucose deprivation and reperfusion caused sp1 transcription factor-mediated release of eCIRP into culture medium, a 4-fold increase in release compared to control conditions [52]. Recently, the Nes-Cre driven neural-specific CIRP deficient mice with CIRP deficiency in both neurons and glial cells alleviated the neuronal apoptosis and inflammatory microglial activation in the context of TBI [53], supporting microglia and neurons as the source of eCIRP. Despite the neuroprotection conferred by iCIRP the deleterious effects of eCIRP prevail, as we and others have shown that CIRP^−/−^ mice in IS, ICH, and TBI models showcase improved outcomes, decreased neuron death and neuroinflammation [50, 53, 54].

### Potential mechanisms of eCIRP in CNS injury

The role of eCIRP as a potent inflammatory mediator is well established in animal models of inflammatory injury, such as hemorrhagic shock and hepatic, renal or mesenteric ischemia-reperfusion models of sterile injury, as well as non-sterile model systems of murine neonatal and adult polymicrobial sepsis [15, 55–64]. These studies provide insights into some potential but not yet proven mechanisms of eCIRP’s role in CNS injury. We and others have reported eCIRP as a major pro-inflammatory mediator in CNS injuries [50, 52–54]. We will discuss the mechanisms for eCIRP’s role in the context of various CNS resident and infiltrating peripheral immune cells subsets. We have included mechanisms which have been shown in CNS models as well as cell-specific mechanisms identified in non-CNS inflammatory diseases, which offer promising avenues for future research in CNS injury.

#### Microglia

Microglia, the tissue resident macrophages of the CNS, are the front-line of inflammatory cells that first sense and respond to stress and injury in the CNS [65–67]. We have shown that eCIRP binds to and signals to microglia acting as DAMP in cerebral ischemic injury as well as with alcohol consumption [16, 17, 50]. Strikingly, brain tissues of CIRP deficient mice demonstrated reduced infarct volume and diminished microglial activation in comparison to wild type mice, when subjected to IS via permanent middle cerebral artery occlusion (pMCAO) [50]. We have shown that eCIRP stimulation of microglia caused elevated TNF-α release leading to neuronal cell injury in cerebral ischemia via TLR4 (45). eCIRP also aggravated neuroinflammation via TLR4 signaling in intracerebral hemorrhage-induced brain injury which supported our findings [54]. Similarly, a TBI study found eCIRP to downregulate histone H3 acetylation resulting in diminished α7nAChR expression and amplifying expression of TNF-α and IL-1β in BV-2 cells in a TLR4-dependent mechanism [53]. In the era of single cell RNA sequencing, microglia have been found to exhibit disease-specific phenotypes, including IS which has a unique and sexually dimorphic transcriptomic profile [68, 69]. It is not yet known how eCIRP influences the microglial transcriptome in CNS injury.

Efferocytosis, or the engulfment of dead and dying cells, is a novel process demonstrated in a variety of disease states including CNS injury and neurodegenerative disease [70–72]. In multiple models of CNS injury, efferocytosis has been shown to be beneficial, with loss of function studies demonstrating poor outcomes correlated with efferocytosis function, and augmentation of efferocytosis resulting in improved outcomes [71, 73–79]. Interestingly, macrophage bacterial phagocytosis is impaired by eCIRP in sepsis [80]. While the targets of phagocytosis, foreign particles and pathogens, and efferocytosis, dead cells, are highly dissimilar, much of the cellular machinery utilized by professional phagocytes involved in reorganization of actin networks, formation of phagocytic cups, internalization, endolysosome trafficking and processing are the same [81, 82]. Based on the above findings in non-CNS contexts, eCIRP could also be a key mediator in microglial efferocytosis in context of CNS injuries.

#### Neurons

eCIRP induced apoptosis in neurons indicated by upregulation in caspase activity and decrease in expression of antiapoptotic protein Bcl-2 in SH-SY5Y neuronal cells [50]. More recently, eCIRP was shown to induce neuronal apoptosis in TBI model via activation of ER stress-related apoptotic pathway [53]. In this study, eCIRP upregulated the expression of p-PERK, GRP78, ATF4 and CHOP in Neuro-2a cells and caused ER-swelling. eCIRP directly triggered the IL-6Rα/STAT3/Cdk5 pathway in neurons, mediated by p25 [49]. p25 is canonically known to form the complex with Cdk5 and result in neuronal apoptosis and tau hyperphosphorylation [83–85]. Further investigation in our lab recently revealed that this pathway was mediated by PLC-dependent IP_3_ activation causing ER stress and subsequent calcium release to activate calpain and induce hyperactive p25 [86]. Dysregulation in calcium and calpain pathway has also been implicated in neuronal damage in the CNS injury further supporting eCIRP’s role in ER stress-mediated neuronal injury [87, 88].

Additionally, there is growing evidence that the tau phosphorylation pathway is activated in CNS injuries [89–94]. Mice subjected to TBI demonstrate increased brain tissue reactivity to AT8, a specific antibody for phospho-Tau, and higher levels of insoluble tau compared to sham controls, with changes persisting up to 6 months post-injury [94]. Tau hyperphosphorylation is also induced in rat models of IS, with significantly elevated levels of 3- and 4-repeat tau and hyperphosphorylated tau detected in the ischemic penumbra, as well as dynamic changes to tau localization from axons to neuron soma [89]. Furthermore, the calpain-p25-Cdk5 pathway has been shown to cause tau phosphorylation in rat IS, supporting that CNS injury induce tauopathy [93]. Phospho-tau levels have been found to correlate with injury severity in brain samples of TBI patients, and serum phospho-tau levels were predictive of poor 3-month outcomes in ICH patients [91, 92]. Due to eCIRP’s activating effect on Cdk5, it is likely that eCIRP initiates neuronal tau hyperphosphorylation in CNS injury, contributing to the immediate severity of the injury, and potentially predisposing CNS injury patients to neurodegenerative disease. Taken together, these findings suggest eCIRP has a significant role in neuron damage and death in CNS injuries.

#### Astrocytes

Under physiologic conditions, astrocytes have a multitude of functions including facilitating synaptogenesis and synaptic function, and maintenance of the BBB [95]. However, under pathologic conditions astrocytes undergo phenotypic changes resulting in the formation of reactive astrocytes that do not perform their physiologic functions [96]. As discussed previously, BBB permeability has multiple deleterious effects in CNS injury, and loss of astrocyte function at the BBB is likely one of the major causes of this dysfunction. Multiple studies have reported that astrocyte foot processes, which form the CNS side of the BBB, swell during injury and inflammation and lose contact with endothelial cells resulting in permeability in the setting of mild to severe TBI and IS [97–99]. Even in spinal cord injury, which does not have a major component of cerebral edema, astrocytes participate in the pro-inflammatory environment by secreting cytokines [100].

Proinflammatory microglia release IL-1α, TNF-α and C1q, which together are required to induce pro-inflammatory A1 astrocytes, which subsequently secrete a neurotoxic factor resulting in neuronal death [96]. Aside from this neurotoxic factor, A1 astrocytes also secrete C3, a complement protein, that can bind to C3aR receptors expressed on microglia to indirectly exert neurotoxic effects [96, 101]. Astrocyte polarization has recently come into the spotlight as a therapeutic target in CNS injury, with evidence that A1 astrocytes promoting secondary injury, and A2 astrocytes promoting recovery [102–104]. In rodent models of TBI and IS, therapeutic interventions to inhibit A1 polarization and improve A2 polarization have consistently shown improved outcomes and enhanced recovery [103–107]. Taken together, the above findings suggest that eCIRP may directly or indirectly affect astrocyte polarization and function, presenting an unexplored and exciting opportunity for future research.

#### Endothelial cells

The effects of eCIRP in CNS pathologies are likely not restricted to direct binding of eCIRP to CNS resident cells. Cytokines, chemokines and DAMPs are known to disrupt the BBB in brain injury [108–114]. BBB dysfunction has been shown to promote brain edema, a potentially deadly outcome of brain injury, and infiltration of immune cells into the CNS, thus amplifying inflammation, after stroke and TBI injuries [67, 115, 116]. The direct role of eCIRP in causing BBB dysfunction in CNS injuries has not yet been elucidated. However, we will summarize what is currently known about eCIRP signaling in endothelial cells in non-CNS injury models. Intravenous injection of eCIRP has been shown to cause endothelial dysfunction and result in lung permeability [59]. In vitro, eCIRP stimulation caused dose- and time-dependent mouse lung vascular endothelial cell (MLVEC) activation via ICAM-1 protein expression and NAD(P)H oxidase activation [59]. Furthermore, eCIRP induced the Nlrp3 inflammasome activation and subsequent pyroptosis in a dose- and time-dependent manner [59]. Circulating eCIRP induced acute kidney injury in vivo upon intravenous injection along with endothelial cell activation which was attenuated in TREM-1 deficient mice [55]. eCIRP also promoted TREM-1-mediated activation of human renal glomerular endothelial cells in vitro, which was attenuated by TREM-1 inhibitory peptide M3 [55]. Thus, eCIRP may be a major mediator of BBB dysfunction, thus amplifying inflammation in brain injury.

#### Neutrophils

Neutrophil extracellular traps (NETs) are antimicrobial extracellular DNA networks that contain proteins that serve to combat pathogens and amplify inflammation. NETs have been demonstrated to activate and injure endothelial cells, inhibit anticoagulation pathways, and trigger coagulation [117–122]. NETs contain many proteins including histones acting as DAMPs to amplify the inflammatory response, and numerous proteases such as matrix metalloproteinases (MMPs), neutrophil elastase (NE), proteinase 3, and cathepsin-G [123]. A neutrophil protease inhibitory cocktail was able to almost entirely reverse neutrophil mediated neurotoxicity in murine tMCAO [124]. Strikingly, eCIRP is sufficient to induce NETosis as evident by elevated NET content in lungs following intratracheal injection, and in vitro treatment of BMDN via the TREM1/ICAM-1/Rho pathway [125, 126]. Furthermore, a recent study investigated eCIRP-mediated NETosis in murine IS, showing that the eCIRP/TLR4/p38 signaling pathway cause NETosis and subsequent BBB permeability and cerebral edema [52]. Targeted inhibition of this pathway at the eCIRP, TLR4 or sp1 level ameliorated cerebral edema [52]. We have recently discovered a novel subset of highly pro-inflammatory neutrophils named antigen-presenting aged neutrophils (APANs) in sepsis, that have yet to be explored in CNS injury [127]. APANs are a phenotypically distinct subset of neutrophils which secrete increased levels of IL-12 to promote IFN-γ production in CD4^+^ T cells, in turn promoting hyper-NETosis [127]. eCIRP exaggerates CNS injury by promoting the toxic effects of neutrophils and NETs in brain.

#### Macrophages

With regards to literature about eCIRP, perhaps the most heavily investigated cell is the peripheral macrophage [15, 80, 128–131]. Despite this, eCIRP’s effect on infiltrating macrophage in CNS injury is not known. While macrophages have been shown to be a significant source of eCIRP, they are also highly sensitive to eCIRP signaling [39]. Much like microglia, macrophage respond to eCIRP stimulus with the release of TNF-α, IL-6 as well as HMGB1 [15]. Interestingly, eCIRP seems to increase expression of TNF-α by binding to both TLR4 and TREM-1 receptors. THP-1, the human monocyte-like cell line, showed a dose-dependent decrease in release of eCIRP-induced TNF-α when treated with increasing doses of an eCIRP/TLR4 interaction inhibitory peptide, termed C23 [63]. In another study utilizing primary peritoneal macrophages, macrophages from TREM-1^−/−^ mice showed attenuated release of eCIRP-induced TNF-α and IL-6 [132]. One lesser studied function of macrophage is the formation of macrophage extracellular traps (METosis), a novel and not yet well understood response of macrophage to overwhelming inflammatory stimuli, similar to NETosis [133]. Given what is known about NETosis in CNS injury, it is likely that macrophage undergo METosis and contribute to inflammatory secondary injury in the CNS. eCIRP stimulation has been shown to signal THP-1 cells and activate caspase-1 and cause GSDMD pore assembly, through which METs are extruded [129].

Moreover, infiltrating macrophages have also been shown to actively participate in efferocytosis which is important in context of CNS injury attenuation and recovery [134]. eCIRP has recently been shown to inhibit phagocytosis in peritoneal macrophages in vitro by nearly 50% [80]. Mechanistically, eCIRP was found to decrease expression of βPIX and its downstream protein Rac1, which is a GTPase known to highly regulate actin reorganization for phagocytosis [80, 135]. It is possible that apart from affecting microglial efferocytosis, eCIRP may also cause continued efferocytosis dysfunction in infiltrating macrophages in CNS injury, representing an opportunity for therapeutic intervention. The complex interplay between eCIRP and peripheral macrophages in CNS injury is not yet fully understood and needs to be further explored.

#### T lymphocytes

Although T cells are less well-studied in the landscape of acute CNS injury research, recent evidence is coming to light that they can indeed worsen such injuries [136–139]. Like other peripheral immune cells, T cells also infiltrate the CNS in the setting of acute insult [140]. Infiltrating CD4^+^ T cells may then differentiate into Th1 cells and promote tissue injury through secretion of pro-inflammatory cytokines including TNF-α, IFN-γ, and importantly IL-2 which can then result in activation of other T cells as well as macrophages [136]. Alternatively. CD8^+^ T cells have a direct cytotoxic effect through the Fas/FasL pathway, and other reports correlating CD8^+^ cell number with poor recovery, complications, and risk of hemorrhage [136, 141, 142]. Similarly, CD8^+^ T cells have been shown to persist in the CNS up to 8 months after injury in murine TBI, and notably pharmacologic or genetic ablation of CD8^+^ T cells improved neurological outcomes suggesting a deleterious role for cytotoxic T cells in CNS injury [139]. In a study on ICH, CD4^+^ T cells were found in perihematomal tissue, expressed a proinflammatory gene signature, and depletion of the CD4^+^ population significantly improved neurological deficit and reduced perihematomal edema [142]. Our group has shown that eCIRP can signal both CD4^+^ and CD8^+^ lymphocytes via TLR4 [143]. Specifically, intravenous eCIRP injection dramatically increased expression of activation markers CD25 and CD69 in splenic lymphocytes of wild type, but not TLR4 deficient mice [143]. eCIRP induced a Th1 related gene signature in CD4^+^ T cells, and increased Granzyme B and IFN-γ expression in CD8^+^ T cells [143]. This suggests a likely role for eCIRP-mediated lymphocyte pathways in CNS injury.

### eCIRP as a prognostic indicator and biomarker of CNS injury

Much like other diseases and injuries, CNS injury treatment and management often depends on the severity of injury. Clinicians use various data to inform their approach to each patient. In IS, the National Institutes of Health Stroke Score (NIHSS) is a widely used assessment tool for neurological deficit in the acute setting that has been shown to correlate with infarct volumes [144]. The modified Rankin scale (mRS) is another tool used to assess stroke severity, however mRS is primarily used as a measure of outcome severity [145]. The NIHSS, in conjunction with other clinical data, is then used in assessing whether the use of thrombolytic therapy is indicated [146]. Similarly, in TBI various clinical data, including acute serum biomarkers, are used to assess severity to inform clinical decisions regarding acute care and predicting long-term outcomes [147–149]. While there is no ultimate biomarker or predictor of acute or long-term prognosis of CNS injury, a promising new avenue of research is the development of computational approaches integrating multi-parametric data to improve predictive value of these data [147, 149–152].

Elevated plasma eCIRP levels have been of potential prognostic value in variety of inflammatory diseases [153–158]. Indeed, eCIRP is an emerging biomarker for CNS injury. Sera from ICH patients showed dramatically elevated concentrations of eCIRP within 24 h from onset [54]. Furthermore, in this study ICH patients with severe neurological deficits, scoring > 2 on the mRS at 3 months after onset, had significantly elevated serum eCIRP levels when compared to patients scoring ≤ 2 [54]. A single-center prospective study in acute IS patients examined serum eCIRP levels from ≤ 1 day to 7 days post-acute IS onset, as a prognostic indicator for IS severity. Serum eCIRP levels peaked early, followed by a gradual decline after 3 days, with a statistically significant positive correlation with infarct volume, NIHSS, and mRS [18]. Additionally, this study found that patients with higher serum eCIRP, on average, have higher mRS 90 days after IS, suggesting that serum eCIRP levels in acute IS may predict outcomes in the chronic timeframe [18]. Finally, similar findings have been demonstrated in TBI patients. In a small study on TBI, serum eCIRP levels were significantly elevated in TBI patients 1-day post-TBI relative to healthy volunteers, and within the TBI cohort eCIRP correlated with TNF-α, IL-1β, neuron-specific enolase, and S100B [53]. Despite the encouraging results of the herein discussed studies, more work is needed to develop serum eCIRP as a broadly generalizable biomarker for CNS injuries, with careful attention to the temporal dynamics of eCIRP levels.

### Future approaches to target eCIRP in CNS injury

Despite continued efforts at developing a neuroprotective therapy for CNS injury, there has been an ongoing failure to translate pharmacologic therapies into clinical practice [159–161]. In patients presenting with signs and symptoms of stroke, alacrity in evaluation, including head imaging is of utmost importance to differentiate and triage IS versus ICH pathology [146, 161]. Once ICH is ruled out, IS patients may receive thrombolytic therapy alteplase, also known as tissue plasminogen activator, the only FDA-approved pharmacologic therapy for IS [146]. In the case of large vessel occlusion, surgical intervention with mechanical thrombectomy is the only other non-supportive or preventative treatment option [146]. In ICH and TBI, there is no direct pharmacologic therapy option, with management limited to addressing secondary consequences of CNS injury including coagulopathy, hemodynamic instability, hypertension, intracranial insults, and prevention of secondary insult [160, 161]. Currently there is no successful immunomodulatory therapy for CNS injuries (IS, ICH, and TBI). Therefore, targeted immunomodulatory therapy is an exciting avenue for novel therapeutics in CNS injury. Multiple inhibitors for eCIRP have shown impressive efficacy in pre-clinical models of a variety of diseases discussed as follows.

C23, a short 15-mer peptide that competitively inhibits eCIRP binding to TLR4/MD2 complex functions by binding to the MD2 co-receptor for TLR4 [15]. Outside the CNS, C23 has been successful in improving outcome measurements in rodent models of sepsis, neonatal sepsis, intestinal and renal ischemia-reperfusion injury, cardiac arrest and resuscitation, acute pancreatitis, hemorrhagic shock, and pulmonary fibrosis [61, 63, 162–167]. In a murine tMCAO model of IS, C23 was effective in reducing BBB permeability as measured by expression of tight junction proteins and extravasation of Evan’s blue, fibrinogen, and dextran into the brain parenchyma [52]. Another study extensively evaluated the effect of a C23-derived peptide termed “Tat-CIRP” in rodent as well as non-human primate models of IS and ICH [168]. Tat-CIRP reduced infarct volume in tMCAO mice and tissue loss in ICH mice and ameliorated behavioral deficits in both injury models [168]. Furthermore, Tat-CIRP was efficacious in a rhesus macaque IS model, by significantly reducing infarct volumes from 24 h to 30 days after ischemia and enhanced sensorimotor function in the primates [168]. Part of the Tat-CIRP beneficial effects were likely due to eCIRP inhibition of TLR4-MD2 binding. Since eCIRP is known to play deleterious role in TBI [53], it is prudent to assess C23 as a neuroprotective therapy in TBI injury as well.

Another potential approach to inhibiting eCIRP is to target its interaction with TREM-1, as TREM-1 is known to play a deleterious role in CNS injury [169–172]. One TREM-1 antagonist, a 17-mer peptide LP17, has been shown to be effective in reducing inflammation, cerebral edema and neurological deficit in pre-clinical ICH [172, 173], IS [169] and TBI [174] murine models. M3, a 7-mer peptide designed by our lab, is a novel inhibitor that specifically targets the eCIRP/TREM-1 interaction and effectively attenuated inflammatory injury in sepsis [132]. Although therapeutic administration of M3 has not yet been explored in models of CNS injury, M3 has shown efficacy in multiple models of sterile inflammation. In hemorrhagic shock, M3 is effective at reducing systemic injury and serum cytokine levels [175]. In both intestinal and hepatic ischemia-reperfusion models, M3 has been effective at reducing histologic injury score, organ-specific injury markers, systemic inflammation, and improving survival [56, 176]. In renal ischemia-reperfusion, M3 reduced serum IL-6, and conferred a survival advantage [177]. A subsequent study from our group demonstrated M3 to attenuate endothelial cell activation caused by eCIRP injection, suggesting that M3 may have the ability to improve BBB integrity in the context of CNS injury [55]. Given the success of LP17 in various preclinical studies on CNS injury as discussed above, M3 is an exciting potential inhibitor of eCIRP/TREM-1 signaling that must be explored. Since M3 is much smaller than LP17, it is likely that M3 can be more easily delivered to the brain parenchyma through passive transport across the BBB.

Peptide inhibitors are not the only therapeutic option to target eCIRP. Micro-RNAs (miRNA) are endogenously expressed small non-coding RNAs that function to silence target mRNA translation via base-pairing interaction with the 3’-untranslated region of cognate mRNAs and subsequent degradation [178]. Importantly, miRNAs are not limited to the intracellular compartment, but can also be released extracellularly, detectable in serum as circulating miRNAs [179]. Our lab found that one miRNA, miR-130b-3p, is elevated in sera of human septic patients and binds to eCIRP with a high affinity *K*_*D*_ = 5.10 *10^− 10^ M [180]. miR-130b-3p reduces eCIRP’s binding affinity to the TLR4/MD2 receptor complex [180]. In vitro, miR-130b-3p inhibited eCIRP-mediated release of TNF-α and IL-6 from macrophages and was beneficial in attenuating inflammation and organ injury in polymicrobial sepsis [180]. However, due to the presence of endogenous nucleases in circulation, our group bio-engineered miR-130b-3p to protect it from degradation thus creating PS-OMe miR130 [181, 182]. PS-OMe miR130 retained the function of miR-130b-3p, reducing macrophage cytokine release, inhibition of eCIRP binding to TLR4/MD2, and high binding affinity to eCIRP [182]. Furthermore, PS-OMe miR130 demonstrated efficacy in hepatic and renal ischemia-reperfusion injury, two models of sterile inflammation, improving systemic and organ specific injury markers, as well as providing a survival advantage [182–184]. While PS-OMe miR130 has not yet been examined in the context of CNS injury, recent literature supports its potential utility. Hence, PS-OMe miR130 is a novel eCIRP inhibitor that may have multiple mechanisms of action to treat CNS injuries.

Our lab has also recently developed another eCIRP inhibitory molecule composed of RNA. iCIRP’s canonical function as an intracellular RNA chaperone protein was leveraged to target eCIRP, which retains iCIRP’s RNA-binding capacity. A_12_ is a bioengineered mimic of the mRNA poly(A) tail featuring terminal phosphorothioate linkages and a 2’-O-methyl ribose modification to prevent degradation by serum RNases [185]. In vitro, A_12_ modulated eCIRP-mediated pro-inflammatory cytokine upregulation and NF-κB activation in macrophages [185]. Furthermore, A_12_ was effective in attenuating levels of serum organ injury markers and cytokines, histologic grade of acute lung injury, and bacterial load in blood and peritoneal fluid of septic mice [185]. The beneficial effects of A_12_ are not limited to sepsis, as it was also successful in an intestinal ischemia reperfusion model demonstrating that treatment with A12 significantly reducing serum IL-6, organ injury markers, lung injury scores, and survival vs. vehicle treatment [186].

Another class of molecules termed “aptamers” are being explored in pre-clinical studies and clinical trials [187, 188]. Aptamers are short single-stranded molecules, composed of either RNA or DNA, that are designed to bind to a specific target [189]. Importantly, aptamers are highly specific for their targets, with a recent study demonstrating a vol Willebrand factor-specific aptamer to be efficacious at reducing VWF activity in acute IS and transient ischemic attack patient blood samples [187]. A TLR4 inhibitory aptamer tested in the rodent tMCAO model of IS, significantly reduced infarct volume and improved neurobehavioral deficits, without toxic effects or significant changes to physiologic measures [188]. Recently, an eCIRP-targeting aptamer was identified and tested in a rodent model of pancreatitis, successfully reducing injury by blocking the eCIRP/TLR4 interaction [190]. Thus, eCIRP-targeting aptamers are another approach that may yield impressive developments in the field of CNS therapeutics.

Finally, we will discuss the most novel approach to suppress eCIRP signaling in CNS injury. Milk fat globule-epidermal growth factor-VIII (MFG-E8) is an anti-inflammatory protein expressed and secreted by phagocytes and binds to externalized phosphatidyl-serine (PS) on apoptotic cells to act as an opsonin for efferocytosis in macrophages and microglia [191–194]. Additionally, MFG-E8 is known to be beneficial in CNS injury [195–199]. Recently, we discovered that MFG-E8 has a high binding affinity with CIRP and developed a novel MFG-E8 derived small peptide eCIRP inhibitor named MOP3 with the goal of opsonizing eCIRP for phagocytic clearance from circulation [193]. MOP3 demonstrated a high binding affinity to both eCIRP and α_V_β_3_-integrin, promoted macrophage internalization of eCIRP in vitro, and significantly ameliorated release of TNF-α and IL-6 [193]. In vivo, MOP3 was protective in murine sepsis models utilizing both adult and neonatal mice, intestinal ischemia-reperfusion injury, and necrotizing enterocolitis, decreasing markers of organ injury and systemic inflammation, and improving survival [57, 193, 200, 201]. Of particular importance, MOP3 administration significantly decreased the concentration of eCIRP in sera of adult and neonatal septic mice, proving that MOP3 performs its function in vivo [57, 193].

We have discussed several inhibitors of eCIRP, their proven mechanisms of action, and preclinical success. Only a few of these therapeutics have been examined in the context of the CNS injury, representing a landscape of opportunity to develop new drug candidates targeting eCIRP.

### Perspectives and future research directions

**Fig. 1:**
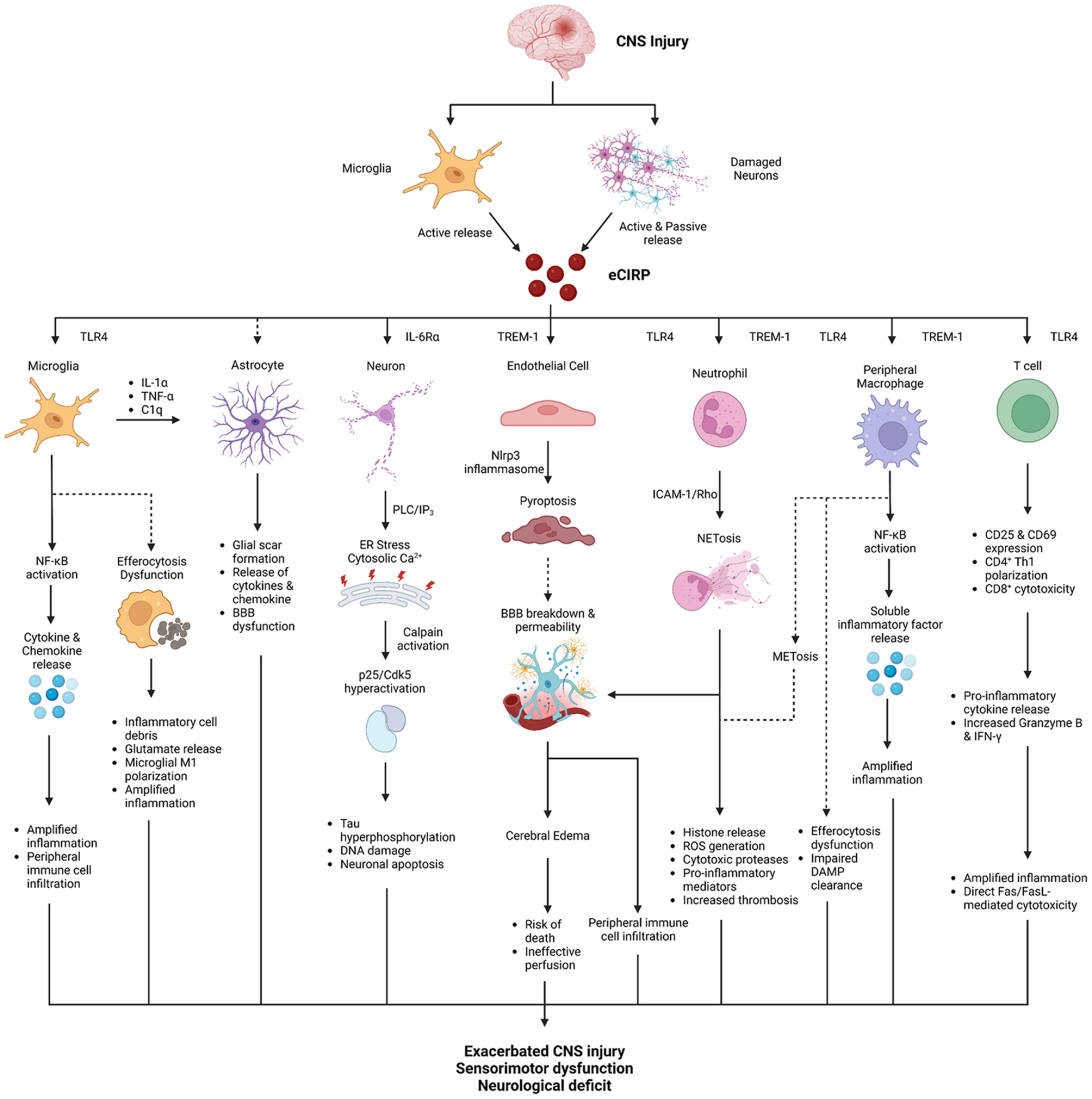
Potential mechanisms of eCIRP in CNS injury. eCIRP is released in CNS injury by microglia and neurons, then signals various CNS resident and infiltrating peripheral immune cells in a deleterious manner to worsen CNS damage. Created with Biorender.com

eCIRP plays a deleterious role in CNS injury via multiple mechanisms which potentially impact a variety of CNS resident and infiltrating cell subsets (Fig. 1) in cell- and receptor -specific manner (Table 1). The best understood mechanism of brain injury is neuroinflammation, but the defective clearance of released DAMPs and injured cells also contributes to exacerbating brain injury [71, 73–75, 78, 202, 203]. As discussed in this review, efferocytosis is a neuroprotective process that limits acute neuroinflammation and enhances recovery from CNS injury. In IS, multiple studies demonstrate the beneficial effects of efferocytosis including reduction in neuron death, reduction in pro-inflammatory cytokines, improved sensorimotor functioning, increased anti-inflammatory microglia/macrophages and decreased infarct volume [74, 75, 78]. Efferocytic dysfunction is also evident in ICH, showing that decreased efferocytosis results in poor neurological recovery, increased iron deposition and reduced anti-inflammatory microglia [73]. Unsurprisingly, analogous phenomena are observed in TBI, showing that when efferocytosis is enhanced, cell death is decreased in the cortex, cerebral blood flow is improved, and BBB permeability is diminished [76].

**Table 1:** Mechanisms of action of eCIRP in various CNS cell types

Cell type	In vitro or Mouse Model (time point)	Receptor	eCIRP effect	Reference
Microglia	BV2 cells (24 h, 48 h)	TLR4	TNF-α, IL-1β release	[16]
	pMCAO (30 h) BV2 cells (20 h, 30 h)		Microglial activation NF-κB activation → TNF-α expression and release	[50]
Neuron	N2a cells and mouse primary neurons (48 h)	IL-6Rα	STAT3 phosphorylation → p25 induction	[51]
	N2a and HT22 cells (48 h)		PLC/IP_3_ activation → ER Ca^2+^ efflux → Calpain activation → p25 induction	[86]
	TBI (1d-28d)		ER stress/swelling, p-ERK/Chop/ Caspase-3-mediated apoptosis	[53]
Endothelial Cell	MLVEC ( < = 8 h), eCIRP *i.v.* (5–24 h)	TREM-1	ICAM-1 upregulation → NAD(P)H oxidase activation → Nlrp3 inflammasome activation → Pyroptosis → Vascular permeability	[59]
	HRGEC (20 h)		IL-6 and other cytokine release	[55]
Neutrophil	BMDN (4 h), eCIRP *i.v.* (5 h, 20 h)	TREM-1	PAD4 activation → NETosis	[125]
	BMDN (4 h), eCIRP *i.v.* (4 h), CLP (sepsis, 4 h)		DAP12/Syk activation → ICAM-1 expression → Rho activation → NETosis	[126]
	tMCAO (24 h)	TLR4	NETosis → Endothelial injury → BBB damage	[52]
	CLP (sepsis, 20 h)		APAN generation → CD4^+^T cell activation → Th1 polarization → IFN-γ-mediated hyper-NETosis	[127]
Macrophage	RAW 264.7 cells and rat and mouse primary peritoneal macrophages (4 h), hemorrhagic shock (4 h)	TLR4	TNF-α, IL-6, HMGB1 release	[15]
	RAW 264.7 cells and primary mouse peritoneal macrophages (24 h), CLP (sepsis, 72 h)	?	STAT3 phosphorylation → Decreased βPIX → Decreased GTP/GDP-Rac1→ less actin remodeling → phagocytosis dysfunction	[80]
	THP-1 cells and primary mouse peritoneal macrophages (3 h, 6 h, 16 h)		Caspase-1 cleavage, H3 citrullination, GSDMD pore formation, METosis	[129]
T lymphocyte	CLP (sepsis, 20 h), eCIRP *i.v.* (20 h), purified mouse CD4^+^ and CD8^+^ T cells (20 h)	TLR4	T cell activation → CD4^+^T cell Th1 response and CD8^+^T cell cytotoxicity	[143]

DAMP clearance is a much less investigated mechanism in CNS injury, however recent work in IS has shown pro-resolving anti-inflammatory microglia and macrophages can effectively internalize and clear DAMPs in the brain, resulting in diminished neuroinflammation, infarct volume, and decreased neurological deficit [202]. Interestingly, eCIRP imparts a pro-inflammatory phenotype in TLR4- and TREM1-dependent manner [204, 205]. On the other hand, chronic eCIRP stimulation can induce endotoxin tolerance skewing macrophages to anti-inflammatory phenotype via IL-6R-STAT3 pathway [131]. To better understand the role of eCIRP in the complex dynamics of microglial heterogeneity, efferocytotic activity and DAMP clearance in the setting of CNS injuries, further studies are needed. Moreover, delineating the mechanisms in detail for eCIRP’s effect on these pathways which resolve brain injuries would be helpful in designing future therapeutic strategies targeting eCIRP.

Aside from microglia and macrophages, eCIRP has been shown to cause ER stress, activation of neurotoxic p25-Cdk5 complex, and apoptosis in neurons [51, 53, 86]. The NLRP3 inflammasome is also activated by eCIRP and causes endothelial dysfunction and apoptosis, potentially implicating the DAMP in BBB dysfunction and microglial pyroptosis [55, 59, 172]. NETosis is another deleterious pathway initiated by eCIRP, with emerging evidence of eCIRP-mediated NETosis causing BBB damage and poor outcomes in IS [52, 125, 126]. eCIRP is known to promote a novel highly inflammatory subtype of neutrophils, aged antigen-presenting neutrophils, which have not yet been assessed in CNS injury [127, 206].

Though mainly neurons and microglia have been investigated, other cells could be source of eCIRP as well. More studies are warranted to deeply dissect the direct effects of eCIRP on individual cell types and receptor-specific mechanisms in CNS injury. Various eCIRP inhibitors have been discussed at length herein (Table 2), spanning from small peptides inhibitors of eCIRP (C23, M3), bio-engineered PS-OMe miR130 derived from an endogenous miRNA, synthetic poly(A) tail A12, opsonic inhibitor MOP3, to modified DNA aptamers [56, 57, 162, 182, 185, 190]. M3, PS-OMe miR130, and MOP3 have shown excellent results in improving sterile inflammation, and warrant exploration as therapeutic agents in the CNS [55, 56, 132, 176, 177, 182–184].

**Table 2:** eCIRP-inhibitors, mechanisms of action, preclinical models, and outcomes

Inhibitor	Mechanism	Model (time point)	Outcome	Reference
C23	Competitive binding to TLR4/MD2	tMCAO (IS, 24 h)	↓ BBB permeability	[52]
		CLP (sepsis, 20 h, 10d survival)	↓ Systemic inflammation ↓ Lung and renal injury ↑ Survival	[162]
		Cecal slurry (neonatal sepsis, 10 h)	↓ Systemic inflammation ↓ Organ injury markers ↓ Lung injury/apoptosis	[163]
		Intestinal I/R (4 h)	↓ Gut and systemic injury	[63]
		Renal I/R (24 h, 7d survival)	↓ Systemic inflammation ↓ Renal histologic injury ↑ Survival	[164]
		Cardiac arrest/ resuscitation (24 h, 7d survival)	↓ Serum cytokines ↓ CNS cytokines ↓ Hippocampal apoptosis ↓ Neurological deficit score ↑ Survival	[165]
		Hemorrhagic shock (4.5 h)	↓ Lung inflammation ↓ Systemic inflammation ↑ Lung endothelial function	[61]
		Acute pancreatitis (72 h)	↓ ROS, pyroptosis, ↓ Inflammation ↓ Pancreatic injury	[166]
		Pulmonary fibrosis (22d)	↓ Fibrosis markers ↓ Lung fibrosis score	[167]
M3	Competitive binding to TREM-1	CLP (sepsis, 20 h, 10d survival)	↓ Systemic inflammation ↓ Organ injury ↓ Lung injury score ↑ Survival	[132]
		Intestinal I/R (4 h, 24 h survival)	↓ Gut/Lung/Serum cytokines ↑ Survival	[56]
		Renal I/R (24 h, 10d survival)	↓ Renal cytokines ↓ Renal injury markers, ↓ Histologic injury/apoptosis ↑ Survival	[177]
		Hepatic I/R (24 h, 10d survival)	↓ Systemic inflammation ↓ Hepatic cytokines ↑ Survival	[176]
		Hemorrhagic shock (4 h)	↓ Systemic inflammation ↓ Lung cytokine expression ↓ Lung injury score	[175]
miR-130b-3p	Competitive binding to eCIRP	CLP (sepsis, 20 h)	↓ Systemic inflammation ↓ Lung cytokines ↓ Lung injury score	[180]
PS-OMe miR130	Competitive binding to eCIRP	Hepatic I/R (24 h)	↓ Systemic inflammation ↓ Liver injury score/apoptosis, ↓ Liver cytokines	[183]
		CLP (sepsis, 20 h, 10d survival)	↓ Serum cytokines/LDH ↓ Lung cytokines ↓ Lung injury score/apoptosis ↑ Survival	[182]
		Renal I/R (24 h, 10d survival)	↓ Renal injury markers ↓ Renal apoptosis ↑ Survival	[184]
A_12_	Competitive binding to eCIRP	CLP (sepsis, 20 h, 10d survival)	↓ Systemic inflammation ↓ Blood/peritoneal bacteria ↑ Survival	[185]
		Intestinal I/R (4 h, 48 h survival)	↓ Systemic inflammation ↓ Gut cytokines/chemokines ↓ Intestinal/lung injury scores ↑ Survival	[186]
XA-CIRP	Competitive binding to eCIRP	Acute pancreatitis (72 h)	↓ Pancreatic injury score ↓ Pancreatic necrosis ↓ Pancreatic RIP-3 expression ↓ CD11b & F4/80 infiltration ↓ Serum amylase and lipase ↓ Serum cytokines	[190]
MOP3	Opsonization/ internalization of eCIRP	CLP (sepsis, 20 h, 10d survival)	↓ Systemic inflammation ↓ Lung injury score/apoptosis, ↓ Lung cytokines ↑ Survival	[193]
		Cecal slurry (neonatal sepsis, 10 h, 7d survival)	↓ Systemic inflammation ↓ Lung/Gut inflammation ↓ Histologic injury/apoptosis ↓ Gut barrier dysfunction ↑ Survival	[57]
		Necrotizing Enterocolitis (NEC, 4d, 5d survival)	↓ Systemic inflammation ↓ Intestinal cytokines ↓ Serum eCIRP levels ↓ NEC histologic injury ↓ Lung injury score ↑ Gut barrier function ↑ Survival	[201]
		Intestinal I/R (4 h, 36 h survival)	↓ Systemic inflammation ↓ Gut/lung injury score ↓ Gut apoptosis ↑ Tight-junction expression ↑ Survival	[200]

The potential for eCIRP as a biomarker for CNS injury is also growing. Serum eCIRP concentration is elevated in IS, ICH, and TBI, suggesting that there is clinical utility to measuring eCIRP to diagnose CNS injuries [18, 53, 54]. Furthermore, in all three of these injuries, eCIRP levels correlate with severity and poor recovery supporting the use of eCIRP as a prognostic indicator to inform clinicians about long-term outcomes of their patients [18, 53, 54]. Mounting evidence suggests that eCIRP is a broadly applicable marker to CNS injury severity, however more investigation is needed to assess its utility as a biomarker in other CNS pathologies as well, and to evaluate its sensitivity and specificity in diagnosis and prognostic indication of CNS injury.

## Conclusion

In summary, eCIRP is a pleiotropic pro-inflammatory mediator, that is receiving growing attention as a critical factor in CNS injuries. The role of eCIRP in many systemic and organ-specific models of inflammation has been well-established, with multiple mechanisms that have not yet been elucidated in the context of the CNS. Future studies are needed to understand the complex and multi-factorial role of eCIRP in the CNS injury, to develop it as a potential therapeutic target, and prognostic biomarker.

## Data Availability

No datasets were generated or analysed during the current study.
